# Comparison of Physiological Responses to Plant-Protein- and Carbohydrate-Based Fishmeal Substitutes in Largemouth Bass (*Micropterus salmoides*)

**DOI:** 10.3390/ani16182969

**Published:** 2026-09-21

**Authors:** Caixia Lei, Jingxing Tian, Xin Lan, Hongmei Song, Jinxin Du, Tao Zhu, Jing Tian, Shengjie Li

**Affiliations:** 1 Key Laboratory of Tropical and Subtropical Fishery Resources Application and Cultivation, Ministry of Agriculture and Rural Affairs, Pearl River Fisheries Research Institute, Chinese Academy of Fishery Sciences, Guangzhou 510380, Chinatianjj@prfri.ac.cn (J.T.);; 2 College of Fisheries and Life Science, Shanghai Ocean University, Shanghai 201306, China

**Keywords:** fishmeal replacement, soybean meal, α-starch, metabolic differences, *Micropterus salmoides*

## Abstract

Developing cost-effective fishmeal alternatives is crucial due to the continuously increasing costs. Carbohydrates are the cheapest alternative, with soybean meal (SM) being the most widely adopted substitute. Nevertheless, few systematic comparisons have addressed their metabolic differences in low-fishmeal feeds. A 60-day rearing experiment examined the physiological responses of *Micropterus salmoides* to fishmeal replaced by SM versus α-starch (ST). Results showed that dietary SM and ST did not alter the intestine/body length ratio or condition factors. SM-fed fish had a significantly higher final body weight, specific growth rate, and carcass index than those fed ST at 5–15% supplementation. High (≥15%) ST increased hepatopancreas and adipose indices, hepatic and muscular lipids, serum triglyceride, cholesterol, tissue glycogen, and hepatic aspartate aminotransferase and alanine aminotransferase activities. The SM groups possessed higher intestinal digestive enzyme activities, while trypsin increased in the ST groups at 15–20% substitution. Histologically, massive hepatic lipid vacuolation occurred in high-level (≥15%) ST groups. Moderate SM thickened the intestinal muscular layers and widened the villi, while ≥15% SM induced severe intestinal morphological damage and upregulated intestinal inflammation-related gene expression. Moreover, 5–15% SM stimulated the mTOR pathway and amino acid absorption gene expression, while suppressing protein degradation-related gene transcription. Higher (≥15%) ATP/AMP ratios were found in high-level SM groups than in their ST counterparts. Importantly, ST exerted a higher protein efficiency ratio at 15% inclusion. This study systematically characterized the physiological disparities between plant-protein and carbohydrate fishmeal alternatives for *M. salmoides*, guiding the reduction of physiological costs from different fishmeal replacement strategies.

## Simple Summary

Fishmeal replacement has been a concern in aquaculture, and it is important for the sustainable development of aquaculture and marine ecology. The study investigated the physiological responses of *Micropterus salmoides* to fishmeal replaced by soybean meal (SM) versus α-starch (ST). The results revealed that dietary substitution with SM yielded superior growth outcomes within the 5–15% inclusion range relative to equal levels of ST. Notably, the adverse effects of SM exceeded those of ST when the substitution level reached 20%, owing to severe intestinal inflammatory responses. ST diets mainly reprogram hepatic lipid metabolism in fish and consequently induce fatty liver symptoms, whereas SM diets primarily damage the physical intestinal structure. Greater attention should be paid to the rational utilization of dietary carbohydrates, as appropriate carbohydrate supplementation (15%) can exert a prominent protein-sparing effect. These findings provide valuable information for the balanced application of plant protein and carbohydrate sources in the feed formulation of *M. salmoides*, which can mitigate the adverse physiological costs induced by different fishmeal replacement strategies.

## 1. Introduction

Proteins are the preferred energy source for fish compared with terrestrial animals [1]. Currently, fishmeal is the top-quality protein source for aquatic animals due to its palatability and digestibility, and is indispensable in many aquatic feeds [2]. Worryingly, global marine catches have plateaued since the mid-2000s, and the sustainability of fishery resources has continued to deteriorate [3]. Over the past 40 years, reducing fishmeal has been a concern in aquaculture, and it is important for the sustainable development of aquaculture and marine ecology.

Currently, fishmeal replacement is predominantly achieved through alternative protein substitutions (e.g., single-cell proteins, insect proteins, and plant proteins) and non-protein energy-saving substitutions mediated by dietary carbohydrates and lipids, whose replacement effects have been extensively studied [4,5,6,7,8]. In practical aquaculture production, soybean meal (SM) is the most commonly used alternative protein. As the most widely used substitute, SM is rich in certain key amino acids [9] and is capable of replacing 20–45% fishmeal [10]. However, it contains several anti-nutritional factors that reduce digestibility [11]. Of greater concern, the large-scale fishmeal replacement by soybean meal in China is restricted by the low self-sufficiency rate (<20%) and considerable supply-demand deficit (≥90 million tons) of soybean meal [12].

Carbohydrates and lipids are cost-effective non-protein energy sources for fish, and their protein-sparing effects are widely accepted [13,14]. Dietary lipids act as carriers of essential fatty acids and play an important role in maintaining cell membrane structure and are precursors for certain bioactive compounds such as prostaglandins, which regulate fish growth and immune function [15,16]. Nevertheless, the high lipid content poses substantial challenges to feed processing and storage. In addition, the toxic byproducts generated by the oxidative degradation of feed oils can adversely affect fish [17,18]. In contrast, carbohydrates have a greater protein-sparing effect than lipids [19]. Carbohydrates can also reduce the pollution of aquaculture water bodies and facilitate feed pellet puffing as they are a non-nitrogen energy source [20]. However, carbohydrates are limited in fish feed due to the inherent high-carbohydrate metabolic disorder in fish, especially carnivorous fish [21]. Fish growth, survival rate, immunity, and tissue structure were negatively affected by high-carbohydrate diets [22,23]. Although some omnivorous and herbivorous fish can adapt to diets containing 40% carbohydrates [24], the recommended dietary carbohydrate level for carnivorous fish is approximately 10% [25,26].

To date, studies have primarily focused on the effects of solitary alternatives at varying levels, effects of different alternative sources, influence of additives on improving the ability of fish to utilize these alternatives, and the effects of different alternative ratios on fish [27,28,29,30,31,32,33]. Interestingly, carbohydrates are generally regarded as unfavorable for fish owing to adverse effects on growth and immunity resulting from their low carbohydrate tolerance, thereby promoting a continuous reduction as far as processing technologies permit in practical production. However, the scientific basis behind this notion still requires further validation. To the best of our knowledge, studies on metabolic disparities among energy-yielding nutrients within fish remain scarce, with few systematic comparisons.

*Micropterus salmoides* is a widely consumed food fish in China and is cultivated nationwide. It has a complete industrial chain covering seedling breeding, aquaculture farming, feed production, logistics and distribution, and product processing. Famous for its tender flesh, negligible intermuscular spines and superior edible quality, the aquaculture production of *M. salmoides* has more than doubled in the past five years, currently exceeding 1 million tons [34,35]. *M. salmoides* is a typical carnivorous fish and has a high demand for protein (48–51%) [36] and a poor acceptance of carbohydrates [22], making feeding costs high, particularly for fishmeal protein. Therefore, finding a cheaper fishmeal substitute is urgently required for this species. Nevertheless, it remains uncertain which is the optimal fishmeal-replacement option between the most widely utilized ingredient (soybean meal) and the low-cost energy source (carbohydrates). Therefore, this study aimed to explore the metabolic differences of *M. salmoides* subjected to fishmeal substitution with plant proteins and carbohydrates to determine the optimal substitute. These results provided a theoretical basis for optimizing feed formulations, reducing breeding expenses, and promoting sustainable aquaculture.

## 2. Materials and Methods

### 2.1. Fish and Feed Preparation

The experimental fish (average weight of 21.22 ± 0.58 g) were obtained from Liang’s Aquatic Seed Industry Co., Ltd. (Foshan, China). After 24 h of fasting, 20 fish were randomly assigned to eight cages (1 m × 1 m × 1.5 m) in triplicate. Eight isonitrogenous and isolipidic expanded diets were formulated by Puhui Feed Co., Ltd. (Jiangmen, China), and the target levels of protein and lipid were 49% and 12%, respectively. Fishmeal was replaced by either plant protein (soybean meal, SM group) or carbohydrate (α-starch, ST group) as the energy source, given the higher digestibility of gelatinized carbohydrate-source ingredients than their raw counterpart [37] (Table 1).

### 2.2. Feeding and Sampling

Each diet was randomly assigned to three cages. Fish were fed to apparent satiation twice daily at 8:00 and 17:00 for 60 days. During the experimental period, dissolved oxygen was maintained at ≥7 mg/L, and natural light was provided. One hour after each meal, 10–15% of the water was replaced. No fish died in the course of this experiment.

Sampling was performed after the fish were deprived of food for 24 h and anesthetized with 2-phenoxyethanol (0.01% *w*/*v*; Sigma-Aldrich, Shanghai, China). The final weight (FW), body length, carcass weight, liver weight, adipose tissue weight (derived from the mesentery), and intestinal length of each fish were measured. The sampled muscle, liver, and intestine (mixed) tissues were rapidly frozen in liquid nitrogen after being washed with pre-cooled phosphate-buffered solution (pH = 7.4, Beyotime Biotechnology Co., Ltd., Beijing, China), and stored at −80 °C. For each cage, six individual fish were used for tissue sample collection. Portions of the liver and intestinal tissues were fixed in 4% paraformaldehyde (Beyotime Biotechnology Co., Ltd., Beijing, China). Another four fish from each cage were used for blood sample collection from the caudal vein. The blood samples were kept at 4 °C for 6 h, and serum was obtained by centrifugation at 2500 rpm for 10 min at 4 °C. The serum was then kept at −80 °C. Fish used for blood collection were also used to obtain liver and muscle samples, and these tissue samples were stored at −20 °C. The hepatopancreas index (HSI), adipose tissue index (ATI), carcass index (CI), specific growth rate (SGR), condition factor (CF), intestine-to-body length ratio (IBL), and protein efficiency ratio (PER) were calculated as follows:

HSI (%) = liver weight × 100/body weight

ATI (%) = adipose tissue weight × 100/body weight

CI (%) = carcass weight × 100/body weight

SGR (%/d) = (Ln final body weight–Ln initial body weight) × 100/days

CF (g/cm^3^) = body weight × 100/body length^3^

IBL ratio (%) = intestine length × 100/body length

PER = (final weight − initial weight) × 100/(feed intake × feed protein content)

### 2.3. Biochemical Analysis

The crude protein and lipid contents of tissues and diets were determined according to AOAC methods [38]. Crude protein was measured via 98% sulfuric acid digestion followed by Kjeldahl analysis and was calculated as N × 6.25. Ether-based Soxhlet extraction was performed to measure the crude lipid content.

### 2.4. Serum Parameters

Commercial kits from Applygen Technologies Inc. (Beijing, China) were used to measure the serum triglyceride (TG), total cholesterol, and high-density lipoprotein cholesterol (HDL-CH) levels. Through sequential enzymatic reactions, TG, cholesterol, and HDL-CH were converted into hydrogen peroxide. Benzoquinone imide was generated after hydrogen peroxide reacted with peroxidase, and its absorbance was quantified at 550 nm using a microplate reader (Multiskan FC, Thermo Fisher Scientific Co., Ltd., Shanghai, China). Low-density lipoprotein cholesterol (LDL-CH) was detected according to the manufacturer’s instructions (Solarbio Science & Technology Co., Ltd., Beijing, China). Hydrogen peroxide was produced after enzymatic reactions. Hydrogen peroxide, 4-aminoantipyrine, and aniline analogs were catalyzed by catalase to form a purple compound, and the absorbance was measured at 546 nm (Multiskan FC, Thermo Fisher Scientific Co., Ltd., Shanghai, China).

### 2.5. Enzyme Assays

Before the formal testing, the frozen samples were homogenized in a pre-cooled 0.9% NaCl solution (*w*/*v*, 1/9) at 4 °C. The supernatant obtained by centrifugation at 4000 rpm for 10 min at 4 °C was used for enzyme activity analysis. All enzyme activities were assayed using their corresponding commercial kits according to the manufacturer’s recommendations (Nanjing Jiancheng Biotechnology Research Institute Co., Ltd., Nanjing, Jiangsu, China). The absorbances for intestinal amylase and trypsin were read at 660 nm and 253 nm, respectively, while intestinal lipase activity was measured by recording fluorescence absorbance at 580 nm. Moreover, hepatic alanine aminotransferase (ALT) and aspartate aminotransferase (AST) activities were assayed by detecting absorbance at 505 nm. The BCA method (Nanjing Jiancheng Biotechnology Research Institute Co., Ltd., Nanjing, Jiangsu, China) was used to determine the protein content of the samples.

### 2.6. Tissue Glycogen Assay

A commercial kit (Solarbio Life Sciences Co., Ltd., Beijing, China) was used to detect hepatic and muscular glycogen contents. The sample (0.1–0.2 g) and 0.75 mL extract solution were incubated for 20 min in a boiling water bath, and then centrifuged at 8000× *g* for 10 min at 25 °C after cooling. The supernatant was used for glycogen measurement. The absorbance of the reaction mixture was read at 620 nm.

### 2.7. Adenosine Triphosphate (ATP) and Adenosine Monophosphate (AMP) Content Determination

Muscular ATP content was measured using a commercial assay kit (Biobox Biotech. Co., Ltd., Nanjing, Jiangsu, China) following the manufacturer’s protocols, and the absorbance was read at 340 nm. Muscular AMP levels were determined using a commercial ELISA kit. The samples were homogenized in 0.9% NaCl solution and centrifuged at 1000× *g* for 10 min. The supernatant was used for detection according to the manufacturer’s instructions (Enzyme-Linked Biotechnology Co., Ltd., Shanghai, China). The color of the reaction product was measured at 450 nm.

### 2.8. Quantitative Real-Time Polymerase Chain Reaction

Total RNA was extracted from the muscle and intestine tissues using TRIzol reagent (Takara Biomedical Technology Co., Ltd., Dalian, China). Following quantification with a nucleic acid quantifier (Thermo Fisher Scientific, Shanghai, China) and quality assessment via 1% agarose gel electrophoresis, cDNA was synthesized using a commercial reverse transcription kit (Takara Biomedical Technology Co., Ltd., Dalian, Liaoning, China) according to the manufacturer’s protocols. Gene amplification was performed in triplicate in 20 μL made up of 25 ng cDNA, 0.4 μL forward primer, 0.4 μL reverse primer, 10 μL 2 × TB Green Premix Ex Taq (Takara Biomedical Technology Co., Ltd., Dalian, Liaoning, China), and sterile ultrapure water. The reaction was initiated at 95 °C for 30 s, followed by 40 cycles of 95 °C for 5 s, annealing at respective temperatures for 30 s, and 72 °C for 30 s (Applied Biosystems, Foster City, CA, USA). The product specificity was determined using a melting curve. The result was calculated using the 2^−ΔΔCt^ method reported by [39,40], and β-actin was selected as the housekeeping gene. The primers [41,42,43,44] and annealing temperatures used are listed in Table S1.

### 2.9. Hematoxylin and Eosin (HE) Staining

The HE staining of liver and intestine tissues was performed according to our previous method [45]. After dehydration in different concentrations (75%, 80%,90%, 95%, and 100%) of anhydrous ethanol, the samples were cleared with xylene (diluted with 100% anhydrous ethanol at a volume ratio of 1:1). Samples were then embedded in paraffin and cut into thin sections. Prior to staining, samples were dewaxed, followed by graded ethanol rehydration. Finally, HE staining was performed according to the manufacturer’s instructions (Beyotime Biotechnology Co., Ltd., Beijing, China). The slices were observed under a ZEISS microscope (AxioScope A1, Carl Zeiss AG, Oberkochen, Germany). To determine the diameter of fat vacuoles, muscular thickness, villi width, and villi height, 60 viewpoints were randomly selected from each group and counted in ImageJ software (v1.54, National Institutes of Health, Bethesda, MD, USA).

### 2.10. Statistical Analysis

The data were first tested for normality using the Shapiro–Wilk test and for homogeneity of variance using Levene’s test. To focus on comparing the metabolic differences of *M. salmoides* in response to fishmeal substitution with SM and ST, an independent-samples *t*-test was performed (PASW Statistics 18, Chicago, IL, USA). *p* ≤ 0.050 was determined to be a significant difference.

## 3. Results

### 3.1. Growth and Biological Parameters

As shown in Table 2, fish fed with SM showed higher FW and SGR than fish fed with ST at 5% to 15% inclusion. No statistically significant differences were observed among the 20% groups. There were no significant differences in the IBL ratio or CF between fish fed the ST and SM diets. The ATI was similar in both the ST and SM groups, ranging from 5% to 10%. However, the ST group exhibited a higher ATI than the SM group at higher addition levels. Similar results were observed for the HSI. Fish fed the SM diet exhibited a higher CI than those fed the ST diet at dietary inclusion levels of 5–15%, and no obvious difference was found between the 20% ST and 20% SM groups. Similarly, PER was not affected by dietary SM or ST, except at the 15% replacement level, where the PER was higher in the ST group than in the SM group.

### 3.2. Muscular and Hepatic Protein and Lipid Contents

The lipid content in the liver and muscle was not affected by a 10% inclusion level in either the ST or SM diet. At higher concentrations, the ST groups exhibited higher hepatic and muscular lipid contents than the SM group. The SM group showed higher muscle protein content than the ST group at all replacement levels (Table 3).

### 3.3. Serum Biochemical Parameters

**Figure 1:**
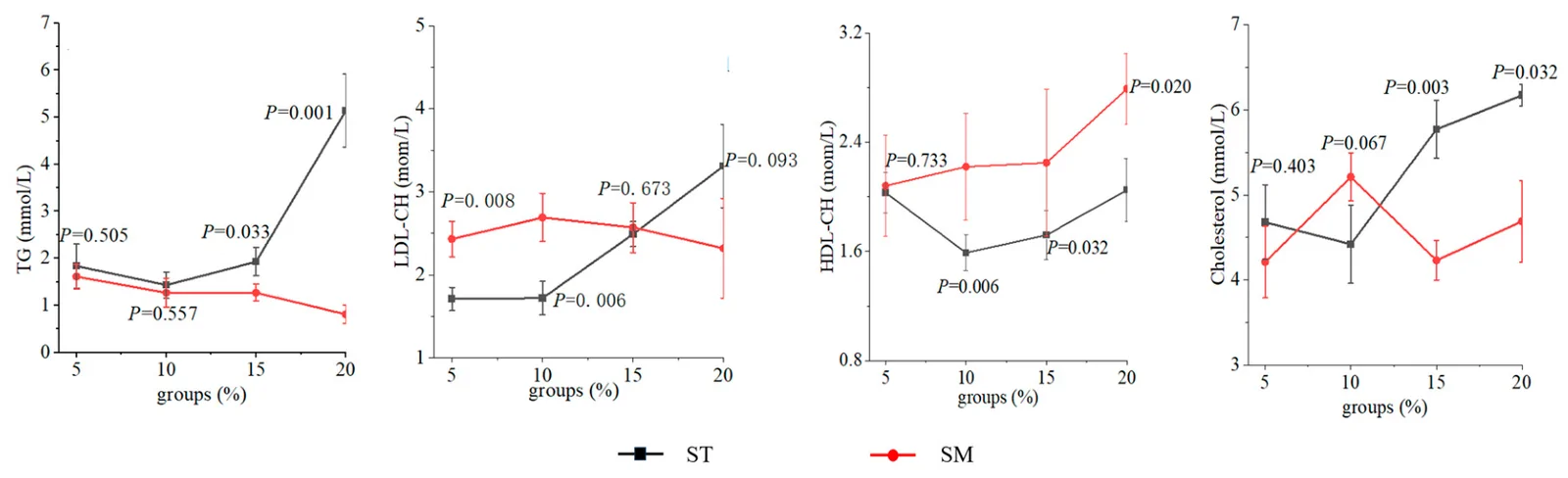
Serum biochemical parameters (means ± SD, n = 3). *p* ≤ 0.050 represents a significant difference.

Serum TG, LDL-CH, HDL-CH, and cholesterol levels are shown in Figure 1. Dietary SM and ST had similar effects on serum TG at 5% and 10% substitution levels, whereas higher serum TG levels were found in the ST group than in the SM group at 15% and 20% levels. At the 5% and 10% levels, the SM group had higher serum LDL-CH levels than the ST group, whereas no significant difference was observed at the 15% and 20% inclusion levels. Regarding HDL-CH, no noteworthy difference was observed between the two groups at the 5% inclusion level, but SM produced higher levels of HDL-CH than ST at higher inclusion levels. Serum cholesterol levels did not differ significantly between the ST and SM groups at a 10% dietary level; however, higher ST inclusion increased cholesterol levels relative to SM.

### 3.4. Enzyme Activity

Hepatic AST and ALT activities showed no remarkable difference between the 5% ST and 5% SM groups, but increased in the ST group compared to the SM group at the other replacement levels.

**Figure 2:**
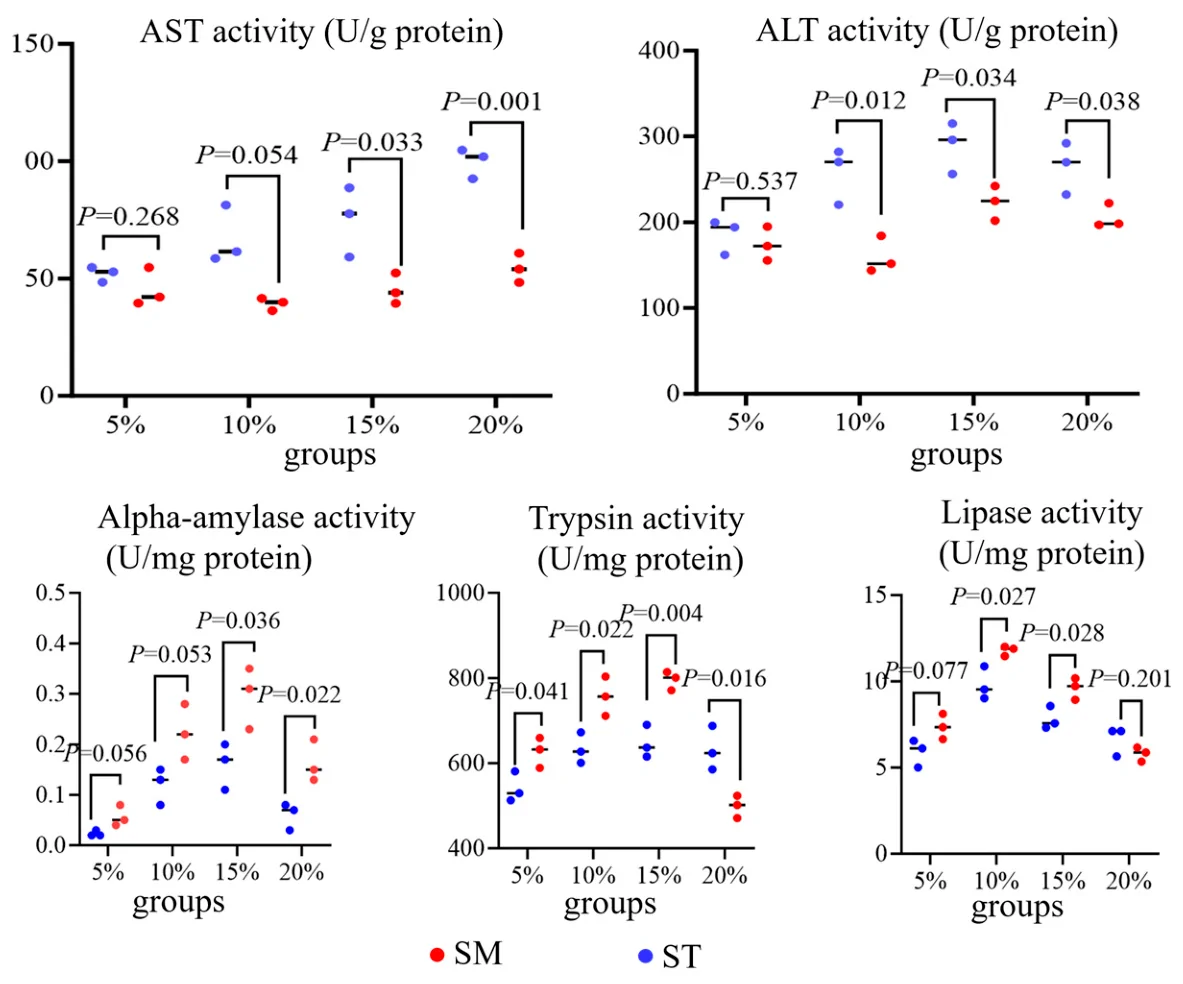
Enzyme activities (n = 3). *p ≤* 0.050 represents a significant difference. (AST and ALT: liver; Digestive enzyme: intestine).

Although insignificant, α-amylase activity was higher in the 5% SM group than in the 5% ST group, and this difference became significant at higher replacement levels. Within the 15% inclusion range, trypsin activity was significantly suppressed in the ST group relative to the SM group, whereas the opposite trend was observed in the 20% group. Lipase activity showed no obvious differences between the ST and SM groups at the 5% and 20% levels, whereas the SM groups presented significantly higher lipase activity than the ST groups at the 10% and 15% levels (Figure 2).

### 3.5. Glycogen Content

**Figure 3:**
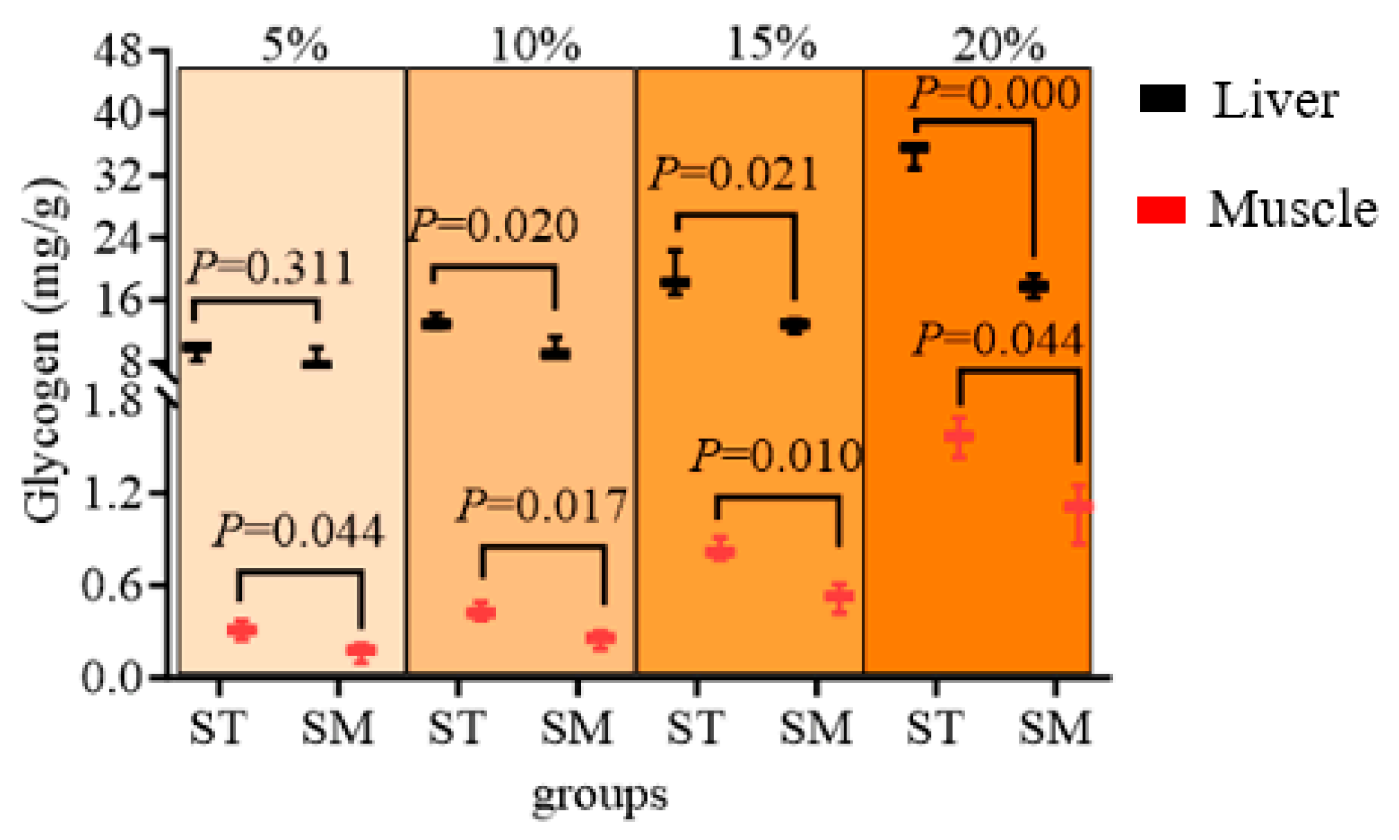
Liver and muscular glycogen content (means ± SD, n = 3). *p ≤* 0.050 represents a significant difference.

Fish in the ST group showed higher glycogen accumulation in the liver and muscles than those in the SM groups. The hepatic glycogen content was not significantly affected by 5% ST or 5% SM (Figure 3).

### 3.6. Muscular ATP/AMP Ratio

**Figure 4:**
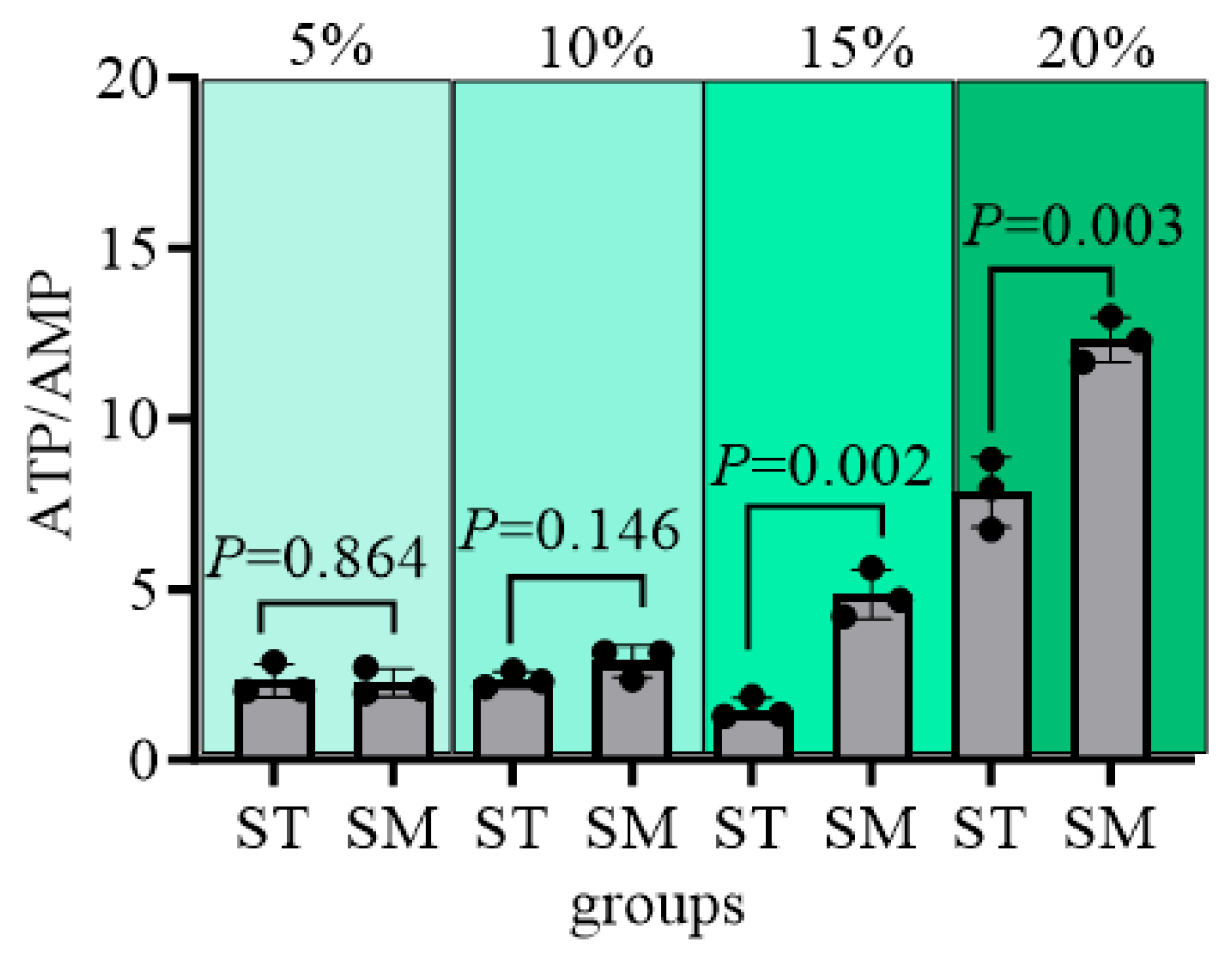
Muscular ATP/AMP ratio (means ± SD, n = 3). *p ≤* 0.050 represents a significant difference.

The muscular ATP/AMP ratios are shown in Figure 4. Equal levels of ST and SM (at 5% and 10% substitution) resulted in no significant differences in the ATP/AMP ratio. However, fish subjected to SM had a higher ATP/AMP ratio than those that consumed ST at 15% and 20% addition levels.

### 3.7. Gene Expression

**Figure 5:**
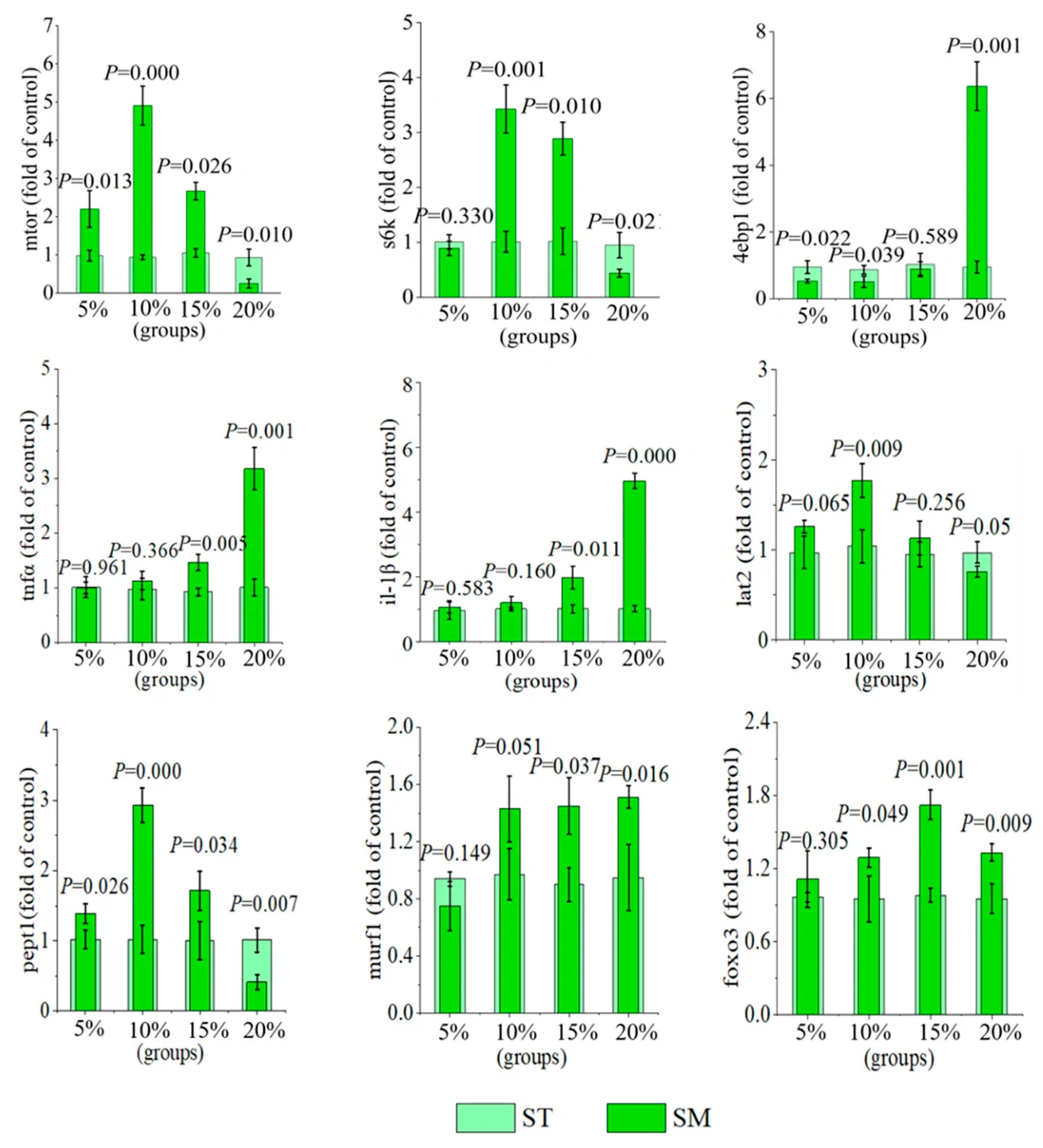
Gene expression (means ± SD, n = 3). *p* ≤ 0.050 represents a significant difference. (mTOR pathway genes, murf1, and foxo3: muscle; inflammation-related genes, pept1, and lat2: intestine).

The SM group exhibited higher mammalian target of rapamycin (*mtor*) expression than the ST group within a 15% inclusion level. The opposite result was observed at the 20% inclusion level. The eukaryotic translation initiation factor 4E-binding protein 1 (*4ebp1*) gene exhibited an opposite trend to *mtor*, while the peptide transporter 1 (*pept1*) gene showed a similar trend to *mtor* in all groups. Dietary 5% ST and 5% SM resulted in similar ribosomal protein s6 kinase (*s6k*) mRNA levels. However, it was higher in the SM group than in the ST group (10–15%). The opposite result was observed at 20% inclusion level. Similarly, the L-type amino acid transporter 2 (*lat2*) gene showed a changing trend. In addition, intestinal tumor necrosis factor alpha (*tnfα*) and interleukin-1β (*il-1β*) shared the same change trend. ST and SM diets had limited effects on gene expression within a 10% inclusion level; however, the expression of the two genes increased in the SM groups from 15% to 20% substitution. Muscle ring finger protein 1 (*murf1*) and forkhead box class o family member transcription factor 3 (*foxo3*) were not affected by ST or SM at the 5% concentration. With an increase in the substitution level, the two genes were enhanced by dietary SM compared with ST (Figure 5).

### 3.8. Organizational Structure Observation

**Figure 6:**
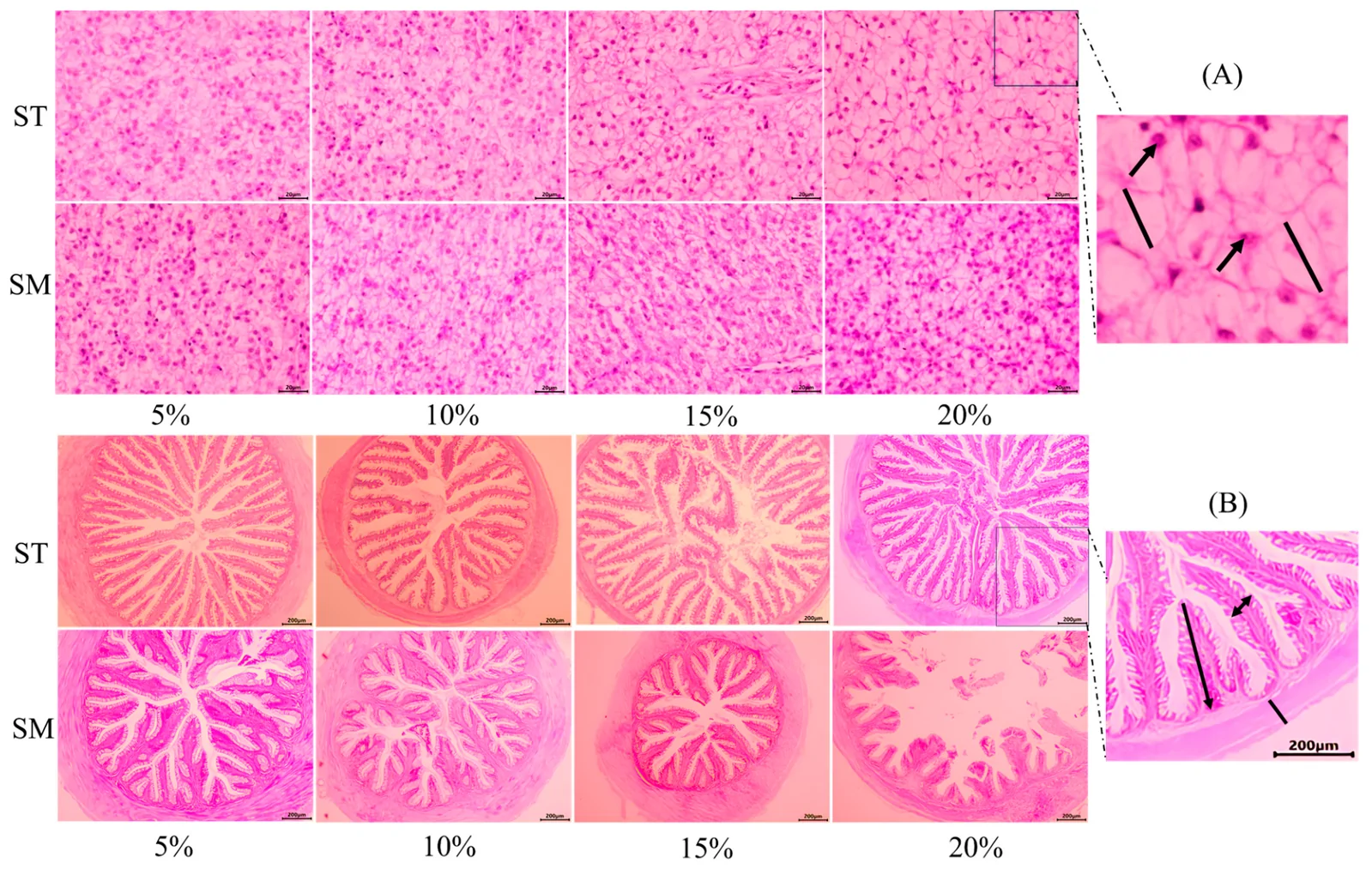
HE staining of liver (**A**) and intestine (**B**) tissues. (**A**) arrow: nucleus; straight line: fat vacuoles; (**B**) unidirectional arrow: villi height; bidirectional arrow: villi width; straight line: myometrial thickness.

**Figure 7:**
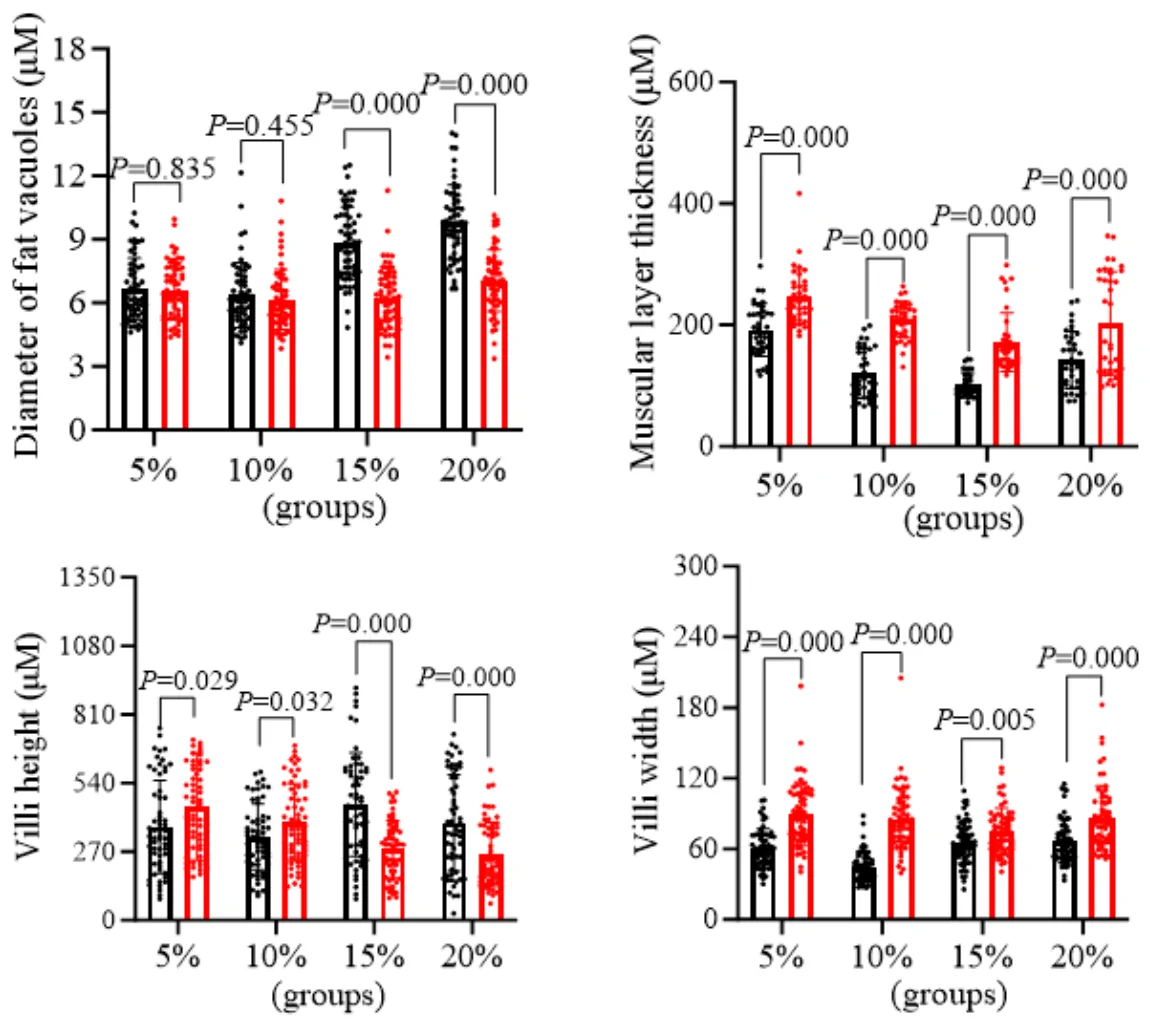
Effect of ST and SM on the morphology of liver and intestinal tissues (n = 60). *p ≤* 0.050 represents a significant difference.

After being fed ST and SM, the livers of the test fish exhibited normal morphology and no evidence of cell infiltration or necrosis was observed. Moreover, the diameter of the fat vacuoles was larger in the 15% and 20% ST groups than in the 15% and 20% SM groups (Figure 6A and Figure 7). Compared to the ST groups, the muscular layer thickness and villi width had increased in the SM group. However, the 15% and 20% SM groups showed decreased villi height compared with the 15% and 20% ST groups, and no significant differences were found at the 5% and 10% levels. Notably, 20% dietary SM caused serious damage to the intestines, leading to villi shedding. The intestinal villi in the SM groups were shorter, thicker, and swollen, accompanied by a curled morphology and disordered branching, compared with the ST groups (Figure 6B and Figure 7).

## 4. Discussion

In this study, the CF and IBL ratio were not significantly different between the ST and SM groups, corroborating the findings of previous studies [46,47]. Compared to ST, fish fed SM exhibited a higher SGR at an inclusion level of less than 20%. Accordingly, higher FW in the SM groups was predictable. This may be due to the feeding habits of *M. salmoides,* which prefer to utilize proteins rather than carbohydrates as the main energy source [48]. In addition, the digestive enzyme activity and intestinal morphology were consistent with the FW and SGR data. For one thing, dietary SM showed higher α-amylase, lipase, and trypsin enzyme activities than the corresponding equal levels of dietary ST at the inclusion level of less than 20%. Though α-amylase remains higher in the 20% SM group than that in the 20% ST group, the damaged intestinal structure offset this beneficial effect. Notably, the trypsin activity was higher in the 20% ST group than in the 20% SM group. More trypsin may be employed to promote protein catabolism for energy production because of the limited carbohydrate utilization ability of fish [21]. Histologically, larger villus width and height correspond to a larger absorptive surface area. A thicker muscular layer facilitates intestinal peristalsis and provides structural support to the intestinal tissue, collectively elevating food digestion efficiency [49,50]. In the present study, the muscular layer thickness and villus width in the SM group were higher than those in the ST group, indicating that soybean meal has a better digestive efficiency than carbohydrates in *M. salmoides*. However, dietary 20% SM exhibited no growth-promoting advantages over 20% ST in terms of SGR and FW, indicating that this SM inclusion surpassed the tolerance limit of *M. salmoides*, which is consistent with the evident intestinal damage in the SM-fed group. Accordingly, villus height was markedly lower in the 15% and 20% SM groups than in the ST group, owing to villous membrane collapse. As a result, higher intestinal *tnfα* and *il-1β* mRNA levels were observed in the 15% and 20% SM groups.

With increasing supplementation levels (15% and 20%), the HSI was significantly higher in the ST group than in the SM group. It has been reported that high-carbohydrate feed caused fatty liver disease in fish [51,52], accompanied by high levels of TG and cholesterol in the liver and blood [53]. In the present study, plasma TG and cholesterol levels, hepatic fat vacuole diameters, and hepatic AST and ALT levels were higher in ST groups than those in the SM groups when the inclusion levels were more than 10%. HDL is primarily responsible for transporting excess cholesterol back to the liver for conversion into bile acids and subsequent excretion. However, high-carbohydrate diets commonly damage the reverse cholesterol transport capacity [54], which explains why lower HDL-CH contents were observed in the 10% to 20% ST groups. LDL is primarily responsible for transporting cholesterol synthesized in the liver to the peripheral tissues. The substitution of SM resulted in lower cholesterol levels in the feed [55], which further stimulated hepatic cholesterol synthesis and led to higher LDL-CH content in the 5% and 10% SM groups. However, the consumption of a high-carbohydrate diet could trigger cholesterol synthesis [56], and this compensatory effect could cause the non-significant differences in the LDL-CH content between the SM and ST groups at the 10% and 20% supplementation levels. The synthesized cholesterol was transported to the adipose tissue by LDL, consequently leading to a higher ATI in the 15% and 20% ST groups.

In the current study, the SM group showed a higher CI than the ST group at the 5–15% inclusion level. Carcass weight is mainly determined by glycogen, TG, and muscle protein levels. Because fish convert excess dietary carbohydrates into glycogen and TG for storage [57], tissue glycogen and crude lipid levels were higher in the ST group than in the SM group, especially at high replacement levels. Collectively, SM may elevate the CI values by stimulating muscle protein synthesis, in line with the higher muscle crude protein levels relative to the ST groups. The mTOR pathway genes involved in muscle protein synthesis were also investigated [58]. The data showed that the SM groups exhibited higher *mtor* and *s6k* expression yet lower expression of the protein synthesis suppressor *4ebp1* than the ST groups at the 5–15% inclusion range, indicating that SM diets are better at promoting muscular protein synthesis. Interestingly, the opposite results were observed in the 20% group. A high-SM diet caused intestinal damage and impaired digestion and absorption, leading to systemic energy deficiency, which has also been reported in *Litopenaeus vannamei* [59]. Under these conditions, the mTOR pathway is inhibited, while the AMPK pathway is activated to synthesize ATP [60]. Correspondingly, the 15% and 20% SM groups have higher ATP/AMP values than those of equal amounts of ST groups. However, no significant difference was observed in the *s6k* expression between the 5% ST and 5% SM groups, indicating that further investigation is required.

Previous studies have demonstrated that moderate carbohydrates have a protein-sparing effect [13]. Here, this effect was reflected in the elevated PER in the 15% ST group compared with the 15% SM group. Unexpectedly, genes *pept1* and *lat2*, which govern amino acid and small peptide absorption, were expressed at significantly lower levels in ST-fed fish than with SM counterparts, within a 15% addition level. Morphologically, dietary SM induces structural damage to the intestine. Specifically, low substitution levels induced stubby, swollen, and curled villi with disorganized branching. These adverse changes were exacerbated at ≥15% inclusion levels, while serious villous shedding occurred at the 20% substitution level. Higher gene expression in these SM groups could be attributed to gene transcriptional compensation, which aligns with previous data reported by Maytorena-Verdugo et al. [61]. In terms of protein catabolic metabolism, the SM group had higher *murf1* and *foxo3* mRNA levels than the ST group at 10–20% supplementation levels. Carbohydrate utilization alleviates energy-driven protein breakdown, which partly explains this phenomenon, while SM-induced enteritis blocks nutrient absorption, and the resultant energy deficiency promotes muscle protein degradation.

## 5. Conclusions

This study systematically compared the metabolic differences in *M. salmoides* induced by fishmeal substitutes containing graded levels of SM and ST. Overall, dietary substitution with SM yielded superior growth outcomes (higher FW and SGR) within the 5–15% inclusion range relative to equal levels of ST, accompanied by elevated muscle protein deposition. Specifically, ST diets mainly reprogram hepatic lipid metabolism in fish and consequently induce fatty liver symptoms, whereas SM diets primarily damage the physical intestinal structure. Remarkably, the adverse effects of SM exceeded those of ST when the inclusion level reached 20%, owing to severe intestinal inflammatory responses. Greater attention should be paid to the rational utilization of dietary carbohydrates, as appropriate carbohydrate supplementation (15%) can exert a prominent protein-sparing effect (higher PER) under the current conditions. These findings provide valuable information for the balanced application of plant protein and carbohydrate sources in the feed formulation of *M. salmoides*, which can mitigate the adverse physiological costs induced by different fishmeal replacement strategies.

Naturally, this study also has several inherent limitations. To produce isonitrogenous and isolipidic experimental diets, the supplementation levels of lipid sources and protein sources were adjusted across treatment groups. Accordingly, the observed differences may arise from these confounding variables, rather than exclusively from SM or ST substitution.

## Figures and Tables

**Table 1:** Composition of experimental diets (SM: soybean meal; ST: α-starch).

Ingredients	Groups (g/100 g)							
	5%		10%		15%		20%	
	ST	SM	ST	SM	ST	SM	ST	SM
Fish meal	45.00	45.00	35.00	35.00	25.00	25.00	15.00	15.00
Meat bone meal	21.03	25.03	20.5	15.53	12.03	25.00	0.00	10.00
Chicken meal	13.00	7.00	22.53	22.00	39.5	21.00	57.53	39.93
Soybean meal	0.00	5.00	0.00	10.00	0.00	15.00	0.00	20.00
Cellulose	6.50	8.40	3.00	7.80	0.00	4.43	0.00	5.60
α-starch	5.00	0.00	10.00	0.00	15.00	0.00	20.00	0.00
Soybean oil	2.00	2.50	2.00	2.50	2.00	2.50	1.00	2.00
Fish oil	3.00	2.60	2.50	2.70	2.00	2.60	2.00	3.00
Premix	2.00	2.00	2.00	2.00	2.00	2.00	2.00	2.00
Lysine	0.03	0.03	0.03	0.03	0.03	0.03	0.03	0.03
Methionine	0.01	0.01	0.01	0.01	0.01	0.01	0.01	0.01
Threonine	0.20	0.20	0.20	0.20	0.20	0.20	0.20	0.20
Antioxidants	0.05	0.05	0.05	0.05	0.05	0.05	0.05	0.05
Choline chloride	0.50	0.50	0.50	0.50	0.50	0.50	0.50	0.50
VC phosphate	0.05	0.05	0.05	0.05	0.05	0.05	0.05	0.05
Carnitine	0.03	0.03	0.03	0.03	0.03	0.03	0.03	0.03
Ca(H_2_PO_4_)_2_	1.50	1.50	1.50	1.50	1.50	1.50	1.50	1.50
Squid paste	0.10	0.10	0.10	0.10	0.10	0.10	0.10	0.10
Total	100.00	100.00	100.00	100.00	100.00	100.00	100.00	100.00
Crude protein (%)	49.64	49.51	49.24	49.61	49.48	49.28	48.60	49.06
Crude fat (%)	11.70	11.39	11.63	11.78	11.78	11.67	11.27	11.87

**Table 2:** ^#^ Growth performance and somatic indexes of experimental fish (means ± SD). *p* ≤ 0.050 represents a significant difference.

Index	ST		SM		*p*	Index	ST		SM		*p*
FW (g)	5%	72.96 ± 8.84	5%	86.08 ± 8.63	0.000	ATI (%)	5%	1.36 ± 0.14	5%	1.53 ± 0.24	0.168
	10%	71.96 ± 7.48	10%	84.76 ± 8.51	0.000		10%	1.45 ± 0.26	10%	1.65 ± 0.31	0.109
	15%	71.85 ± 6.76	15%	80.76 ± 8.06	0.000		15%	1.56 ± 0.37	15%	1.33 ± 0.21	0.054
	20%	64.27 ± 8.99	20%	72.38 ± 8.21	0.187		20%	1.71 ± 0.29	20%	1.39 ± 0.26	0.003
PER	5%	1.59 ± 0.03	5%	1.62 ± 0.06	0.476	IBL ratio (%)	5%	0.79 ± 0.05	5%	0.80 ± 0.05	0.645
	10%	1.73 ± 0.05	10%	1.68 ± 0.05	0.233		10%	0.80 ± 0.06	10%	0.84 ± 0.09	0.205
	15%	2.03 ± 0.06	15%	1.59 ± 0.04	0.000		15%	0.83 ± 0.05	15%	0.82 ± 0.09	0.840
	20%	1.48 ± 0.04	20%	1.45 ± 0.035	0.954		20%	0.79 ± 0.09	20%	0.85 ± 0.10	0.066
HSI (%)	5%	0.88 ± 0.14	5%	0.95 ± 0.20	0.363	SGR (%/d)	5%	2.14 ± 0.11	5%	2.47 ± 0.17	0.027
	10%	1.13 ± 0.23	10%	0.98 ± 0.22	0.068		10%	2.12 ± 0.17	10%	2.43 ± 0.19	0.025
	15%	1.45 ± 0.28	15%	0.86 ± 0.15	0.000		15%	2.12 ± 0.21	15%	2.32 ± 0.19	0.044
	20%	1.67 ± 0.27	20%	0.80 ± 0.17	0.000		20%	1.96 ± 0.15	20%	2.20 ± 0.25	0.056
CI (%)	5%	92.26 ± 0.91	5%	93.09 ± 0.72	0.005	CF (g/cm^3^)	5%	2.43 ± 0.18	5%	2.34 ± 0.13	0.099
	10%	92.49 ± 0.91	10%	93.22 ± 0.82	0.012		10%	2.51 ± 0.18	10%	2.48 ± 0.20	0.678
	15%	92.56 ± 0.94	15%	93.21 ± 0.88	0.027		15%	2.51 ± 0.24	15%	2.42 ± 0.27	0.260
	20%	92.35 ± 1.11	20%	92.85 ± 0.71	0.159		20%	2.41 ± 0.19	20%	2.41 ± 0.17	0.861

^#^ No fish died in the course of this experiment, and 20 fish per cage were included in the statistical analysis.

**Table 3:** The proximate composition in the muscle and liver (g/kg dry weight) (means ± SD, n = 3).

		Lipid		Protein
		Liver	Muscle	Muscle
5%	ST	383.91 ± 16.68	22.57 ± 3.33	890.44 ± 17.41 b
	SM	394.58 ± 26.29	21.46 ± 0.99	922.31 ± 17.60 a
10%	ST	412.84 ± 12.31	26.53 ± 2.37	897.77 ± 20.81 b
	SM	384.54 ± 17.93	22.21 ± 2.67	934.59 ± 14.94 a
15%	ST	459.73 ± 19.08 a	33.35 ± 1.94 a	900.29 ± 24.67 b
	SM	421.02 ± 10.88 b	24.93 ± 1.81 b	926.52 ± 18.75 a
20%	ST	500.13 ± 18.47 a	38.83 ± 0.26 a	897.63 ± 15.73 b
	SM	456.67 ± 19.06 b	28.99 ± 1.24 b	927.19 ± 24.89 a

Different letters mean significant differences (*p* ≤ 0.050).

## Data Availability

The data will be made available on request.

## Supplementary Materials

The following supporting information can be downloaded at: https://www.mdpi.com/article/10.3390/ani16182969/s1, Table S1: Primers used for qRT-PCR [41,42,43,44].
