# Supplementary material for: Comparison of Physiological Responses to Plant-Protein- and Carbohydrate-Based Fishmeal Substitutes in Largemouth Bass (Micropterus salmoides)
Source: Animals (Basel). 2026 Sep 21;16(18):2969. doi: 10.3390/ani16182969 (PMC13603310; doi:10.3390/ani16182969)
Supplement: Supplementary file 1 [file animals-16-02969-s001.zip › animals-4524094-supplementary.pdf]

Table S1. Primers used for qRT-PCR

Gene name	Forward (5’-3’)	Reverse (5’-3’)	Annealing temperature	Reference
β-actin	TCACAGTCCTCCTAAGCCGA	GGCCCATACCAACCATCACA	60 ℃	[41]
tnfα	CGTGAACCAGGAGGAAGACC	CGTCAGCCTGGATAGACGAC	60 ℃	[41]
il-1β	CTGAGCGACCGCAGTAAGAA	TCACTTTGTGGGTGCTGTGT	60 ℃	[41]
mtor	GACGGCTTGGTACAACCTGA	CACTGGCACAGGTTTTCGTG	58 ℃	[42]
s6k	TCCAGAGACTCGTGACACCT	AGCTTGGCATACTCTGAGGC	57 ℃	[42]
4ebp1	GGAGGGTGACCATCAACGAC	CTGGAATGTTGGGCAGACCT	59℃	[42]
murf1	GGATAGCCTCGAGAAGCAGC	AACCTCAAACCGGCAGGTAG	59 ℃	[42]
foxo3	AGTAGCAACAGCCCCATAGC	GGTTATCAGGTCGGCGTAGG	57 ℃	[42]
pept1	CCTATTTGCCTCGCTTTTGGTTGC	CATTAACCTTCGCCGTGAATTGGG	60 ℃	[43]
lat2	GGTGACCACAGGGATAGAGATG	TTGCTTACGGAGGCTGGAACTT	57 ℃	[44]
